# Effects of immersion bathing in *Lactobacillus plantarum* CLY-05 on the growth performance, non-specific immune enzyme activities and gut microbiota of *Apostichopus japonicus*

**DOI:** 10.1371/journal.pone.0315780

**Published:** 2024-12-27

**Authors:** Bin Li, Jinjin Wang, Jianlong Ge, Meijie Liao, Xiaojun Rong, Jinyan Wang, Yingeng Wang, Zheng Zhang, Chunyuan Wang, Yongxiang Yu

**Affiliations:** 1 Key Laboratory of Sustainable and Development of Marine Fisheries, Ministry of Agriculture and Rural Affairs, Yellow Sea Fisheries Research Institute, Chinese Academy of Fishery Sciences, Qingdao, PR China; 2 Laboratory for Marine Fisheries Science and Food Production Processes, Qingdao National Laboratory for Marine Science and Technology, Qingdao, PR China; Tanta University Faculty of Agriculture, EGYPT

## Abstract

In order to study the optimal use of *Lactobacillus plantarum* in sea cucumber (*Apostichopus japonicus*), 49 days feeding trial was conducted to determine the influence of immersion bathing in different concentrations of *Lactobacillus plantarum* CLY-05 on body weight gain rate and non-specific immune activities. The potential effect of CLY-05 on gut microbiota was also analyzed during the immersion bathing at the optimum concentration. The results showed that the body weight growth rate of all bathing groups was higher than that of control. The highest specific growth rate (4.58%) and weight gain rate (25.35%) was achieved at the bacterial concentration of 1×10^3^ CFU/mL. The activities of non-specific immune enzymes (ACP, AKP, SOD and LZM) of all bathing groups increased after immersion bathing, and the enzyme activities of groups bathed with the bacterium at 1×10^3^ and 1×10^4^ CFU/mL reached the highest. Therefore, 1×10^3^ CFU/mL was considered as the optimum concentration of *L*. *plantarum* CLY-05 for *A*. *japonicus* pond culture. The results of gut microbiota analysis showed that the gut microbiota changed with the addition of *L*. *plantarum* CLY-05, and the richness and diversity of the gut microbiota peaked on day 14 and day 21, respectively. The correlation analysis revealed that the non-specific immune enzyme activities were significantly correlated to some gut bacteria (in the phyla Proteobacteria, Firmicutes) after immersion bathing in *L*. *plantarum* CLY-05. These findings provide the theoretical foundation for probiotic application in sea cucumber farming.

## 1. Introduction

The sea cucumber *Apostichopus japonicus* is naturally distributed in the Western North Pacific Ocean and is one of the most valuable sea foods worldwide. Sea cucumber is rather prevalent among the customers because it’s superior nutrition, health care and anti-cancer effects, which is a kind of marine organism with a homology of medicine and food [1]. The aquaculture sector is exploding due to the rapid demand for sea cucumbers, and production is rising every year. According to official statistical data from China, the aquaculture area for sea cucumbers has increased to 2.89×10^5^ hm^2^, with an annual production of up to 2.92×10^5^ t in 2023 However, the rapid development of the *A*. *japonicus* industry has led to emerging issues such as germplasm degradation and a decline in seedling survival rates. At the same time, the aquaculture industry of sea cucumbers is plagued by severe illnesses brought on by numerous pathogens, including *Vibrio splendidus*, *Vibrio alginolyticus*, and others [2–4]. Antibiotics and chemicals were once widely used to treat illnesses, but their extensive usage is now becoming progressively limited because of potential environmental risks include the growth of pathogens that are resistant to antibiotics, environmental contamination, and residue buildup in seafood [5]. Therefore, Probiotics have gained increasing interest in aquaculture as efficient and environmentally acceptable alternatives to antibiotics and pesticides.

A type of common gram-positive bacteria called lactic acid bacteria can produce phenyl lactic acid, cyclic dipeptides, H_2_O_2_, antibacterial peptides, and other substances during the metabolic process to prevent pathogens and spoilage bacteria from growing and reproducing in aquaculture products [6, 7]. Now the use value of lactic acid bacteria is attracting increasing attention that it may help an animal’s digestion and increase its ability to resist disease [8, 9]. It was discovered that *Lactobacillus plantarum* supplementation diet significantly improved the growth rates and α-amylase and trypsin activities of the liver in zebrafish [10]. Tseng et al. [11] found non-specific immunity, immune-related gene expression, and disease resistance of white shrimp (Penaeus vannamei) were significantly increased by feeding the fodder containing 10^5^ CFU/pre shrimp of *L*. *plantarum*. for 14~2l days. However, the use of lactic acid bacteria in sea cucumber production is restricted, and there is not much understanding about how it affects the gut microbiota. Our team’s previous research found that a local probiotic strain *(L*. *plantarum* CLY-05) isolated from the sediment of *A*. *japonicus* farming ponds had a strong inhibitory effect on the growth of *Pseudoalteromonas nigrifaciens* and *V*. *splendidus*. However, the ideal application dosage of *L*. *plantarum* CLY-05 and its potential impact on sea cucumbers remain unknown. Therefore, the effects of different concentrations of *L*. *plantarum* CLY-05 on sea cucumber growth, non-specific immune enzyme activities, and intestinal microbiota were studied in this research in order to determine the best dosage of lactic acid bacteria to add to the *A*. *japonicus* culture and how that would affect the organism’s daily activities. Our findings would serve as a basis for the sensible application of the probiotic *L*. *plantarum* CLY-05 in the breeding of *A*. *japonicus*.

## 2. Materials and methods

### 2.1 Experimental design

Sea cucumber juveniles in good health, weighing 8.7 ± 0.6 g on average, were purchased from a culture company in Qingdao, China. In four plastic containers (83 cm × 64 cm × 60 cm) filled with clean seawater, the sea cucumbers were acclimated for three days. The CLY-05 strain, which was defined in our prior investigation, was screened out of *A*. *japonicus* farming ponds for use in the experiment. By analyzing the 16S rDNA sequence, the CLY-05 strain was identified as *L*. *plantarum*, and the safety test confirmed that it did not cause viscera ejection, disease symptoms, or mortality of sea cucumber. *L*. *plantarum* CLY-05 bathing concentrations of 1×10^2^, 1×10^3^, 1×10^4^, 1×10^5^, 1×10^6^ and 1×10^7^ CFU/mL were used in the experimental groups. Each group consists of three duplicates, each with 60 sea cucumbers chosen at random. Three tanks were utilized as the control group simultaneously, but none of them contained *L*. *plantarum* CLY-05. The experiment was done in 35 L of seawater in plastic tanks. The temperature was kept at 16 ± 2°C, pH at 7.6–8.2, dissolved oxygen at 7±1mg/L for the duration of the experiment, which lasted 49 days, and 1/5 of the seawater’s volume was replaced daily. After replacing the sea water, *L*. *plantarum* CLY-05 bacteria were added. The feed was given to the sea cucumbers at 1% of their overall weight with sea mud and seaweed powder [12].

Every seven days, three individuals from each group were randomly selected, intestinal contents used for microbiota analysis and coelom fluids used for for non-specific immune enzyme activities analysis. Using a disposable syringe, the coelom fluids were removed, then centrifuged for 10 min at 5,000 rpm and 4°C. After that, the supernatants were put into sterile centrifuge tubes so that the activity of non-specific immune enzymes could be measured. The sea cucumber’s intestines were put in cryopreserved tubes and kept in a refrigerator at -80°C to facilitate a later study of the microbiota [12].

### 2.2 Growth determination of *A*. *japonicus*

Every *A*. *japonicus* was weighed on days 0 and 49, and the average weight for each group was determined. The Specific growth rate (SGR) and body weight gain rate (WGR)were calculated as follows:

$$SGR\left(\%\right)=\frac{Wt-W0}{49}\times 100\%$$

$$WGR\;(\%)=\frac{Wt-W0}{W0}\times 100\%$$

*W*_*0*_, the initial body weight,

*W*_*t*_, the final body weight.

### 2.3 Measurement of non-specific immune enzyme activities

We chosen the superoxide dismutase (SOD), enzymes acid phosphatase (ACP), lysozyme (LZM) and alkaline phosphatase (AKP) to depict the non-specific immune enzymes. The enzyme activities of coelom fluids were assessed spectrophotometrically using enzyme activity test kits (A001-3, A060-2, A050-1, A059-2, Nanjing Jiancheng, China) following the manufacturer’s instructions.

### 2.4 Analysis of gut microbiota structure

The E.Z.N.A. soil DNA Kit (Omega Biotek, U.S.) was utilized to extract total genomic DNA from intestinal tissues. By denaturing at 95°C for 2 min, using 25 cycles of denaturing at 98°C for 10 s, annealing at 60°C for 30 s, extending at 72°C for 30 s, and extending again at 72°C for 10 min, the hypervariable region V3–V4 of the 16S rRNA gene was amplified by PCR. Primers 341F (5’-CCTACGGGNGGCWGCAG-3’) and 806R (5’-GGACTACHVGGGTATCTAAT-3’) were used for amplification. The amplified products were purified and sequenced on an Illumina Hiseq 2500 platform by PE250 [12].

To obtain high quality clean readings, raw data that contained adapters or low quality reads was filtered. Using FLSAH (v.1.2.11) [13], paired end clean reads were combined as raw tags with a minimal overlap of 10 bp and 2% mismatch error rates. The high-quality clean tags were then obtained from the raw tags using the QIIME software program (v.1.9.1) [14]. In order to obtained a set of effective tags, identify and eliminate chimeric sequences, the tags were compared to the reference database (http://drive5.com/uchime/uchime_download.html) using the UCHIME method (http://www.drive5.com/usearch/manual/uchime_algo.html) [15]. the effective tags were sorted into operational taxonomic units (OTUs) using the UPARSE pipeline (v.9.2.64). A naive Bayesian model based on Greengenes Database (https://www.arb-silva.de/) [16] and RDP classifier (Version 2.2) [17] was used to classify the representative sequences into species. Using the QIIME (v.1.9.1), the Chao1, Ace, Shannon and Simpson index were determined.

### 2.5 Correlation analysis between enzyme activity and OUT abudance

We used the Cloud platform (https://international.biocloud.net) to analyse the relationship between OTU abundance of gut and enzyme activity of coelom fluids. Greengenes database (https://www.arb-silva.de/) were used to assign OTUs chosen and phyla analysis.

### 2.6 Statistical analysis

Data were expressed as mean ± standard deviations (SD). One-way analysis of variance (ANOVA) was performed to determine the significance of difference among groups. Duncan’s multiple range test was used to compare the significance of difference among treatments. Statistical analyses were performed using SPSS 17.0.

## 3. Results

### 3.1 Growth performance

For all groups, there was no mortality seen. In Table 1, the *A*. *japonicus* body specific growth rate and weight gain rate for each experimental group is listed. The results demonstrated that the SGR and WGR of each experimental group was higher than the control group’s. In comparison to other groups, the SGR and WGR of the 1 × 10^2^ CFU/mL, 1 × 10^3^ CFU/mL and 1 × 10^4^ CFU/mL groups was substantially greater (*p* <0.05). The 1 × 10^3^ CFU/mL group’s growth rate had the highest SGR(4.58%) and WGR (25.35%).

**Table 1:** Growth performance of sea cucumber at different concentrations of *L*. *plantarum* CLY-05.

Groups (CFU/mL)	Initial body weight (g)	Terminal body weight (g)	Specific growth rate (%)	Weight gain rate (%)
Control	8.70±0.13 ^a^	10.59±0.16 ^a^	3.86±0.06 ^c^	21.72±0.05 ^c^
1×10^2^	8.50±0.15 ^a^	10.62±0.18 ^a^	4.32±0.05 ^b^	24.89±0.25 ^a^
1×10^3^	8.84±0.16 ^a^	11.09±0.21 ^a^	4.58±0.10 ^a^	25.35±0.14 ^a^
1×10^4^	8.52±0.16 ^a^	10.67±0.20 ^a^	4.39±0.09 ^ab^	25.25±0.09 ^a^
1×10^5^	8.70±0.15 ^a^	10.63±0.21 ^a^	3.95±0.12 ^c^	22.24±0.36 ^c^
1×10^6^	8.71±0.13 ^a^	10.64±0.15 ^a^	3.95±0.06 ^c^	22.23±0.33 ^c^
1×10^7^	8.63±0.02 ^a^	10.68±0.01 ^a^	4.19±0.05 ^b^	23.78±0.33 ^b^

Note: The different superscript letters within the same column mean significant differences (*P* < 0.05).

### 3.2 Non-specific immune enzyme activities

**Fig 1:**
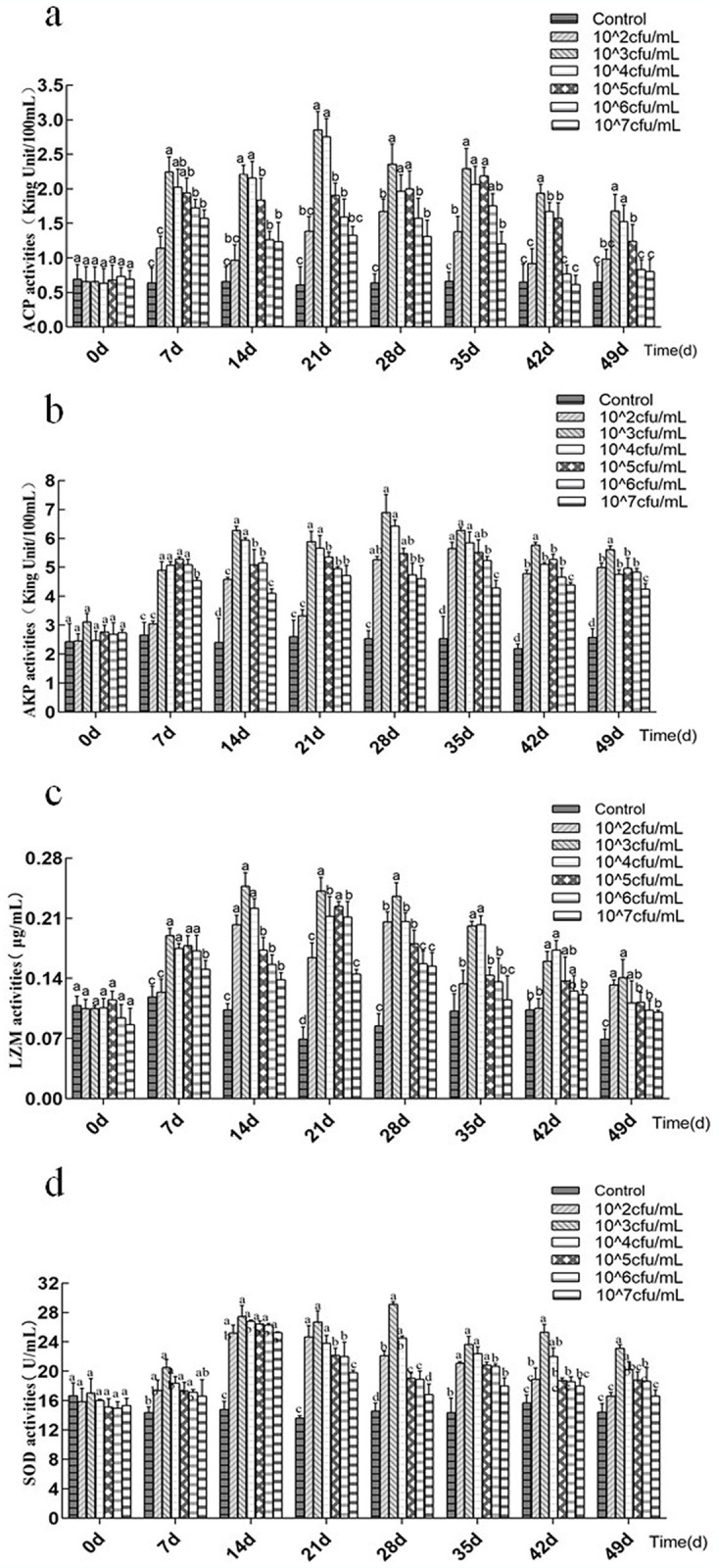
The effect of different concentrations of *L*. *plantarum* CLY-05 on the enzyme activities. (a)activity of ACP, (b) activity of AKP, (c)activity of LZM, (d)activity of SOD.

In order to study the effects of immersion bathing concentrations of *L*. *plantarum* CLY-05 on *A*. *japonicus*, we analyzed the enzyme activity of *A*. *japonicus* in different groups (Fig 1). All immune enzyme activity had no discernible variation between groups at experiment’s initial day. All non-specific immune enzyme activities of experiment groups typically increased at the beginning and reduced at the end of the *L*. *plantarum* CLY-05 treatment, but they were consistently greater than the control throughout. ACP activity increased in the 1 × 10^3^ and 1 × 10^4^ CFU/mL groups on day 21, peaking at a level that was significantly higher than that of the other groups (Fig 1a; *P < 0*.*05*). The AKP activity was consistently higher, with the highest activity occurring on day 28 in the 1×10^3^ CFU/mL and 1 × 10^4^ CFU/mL groups (Fig 1b). From day 14 to day 28, the LZM activity of the 1 × 10^3^ CFU/mL group peaked, and on day 28, it was notably higher than that of the other groups (Fig 1c; *P < 0*.*05*). On day 28, the SOD activity in the 1×10^3^ CFU/mL group peaked and was considerably higher than that in the other groups (Fig 1d; *P < 0*.*05*). These findings demonstrated that the addition of *L*. *plantarum* CLY-05 increased the non-specific immune enzyme activities of *A*. *japonicus*, with the strongest effects being seen in the 1×10^3^ and 1×10^4^ CFU/mL groups.

### 3.3 Gut microbiota analyses

**Fig 2:**
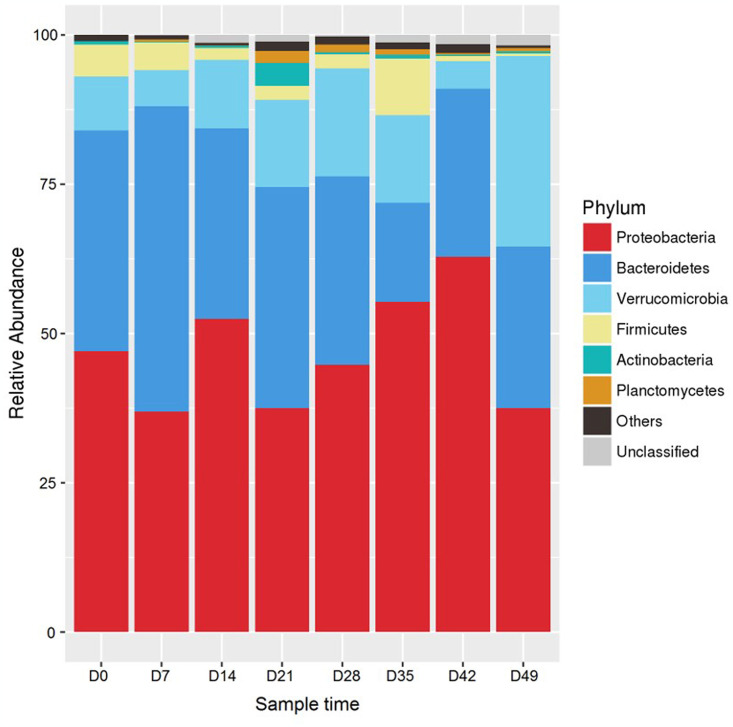
Relative abundances of the gut microbiota at the phylum level in sea cucumber in 1×10^3^ CFU/mL group.

The optimal concentration of *L*. *plantarum* CLY-05 for *A*. *japonicus* pond culture was determined to be 1 × 10^3^ CFU/mL based on growth performance and enzyme activities. After the immersion bathing experiment, 16S rRNA sequencing analysis was used to assess the impact of *L*. *plantarum* CLY-05 in different immersion bathing time. Through the analysis of intestinal flora structure characteristics of 8 time nodes, it was found that the bacteria detected in 24 samples belonged to 18 phyla, 36 classes, 68 orders, 131 families and 211 genera. The top 10 microflora in each group at phylum level were selected to construct the intestinal flora abundance map of *A*. *japonicus* (Fig 2). The results showed that the three phyla with the highest relative abundance in each group were Proteobacteria, Bacteroidetes and Verrucomicrobia, and the relative abundance on 0d, 7d, 14d, 21d, 28d, 35d, 42d and 49d were 47.02%, 36.95%, 9.04%; 36.96%, 51.08%, 6.07%; 52.43%, 31.95%, 11.47%; 37.52%, 37.02%, 14.59%; 44.74%, 31.57%, 18.11%; 55.28%, 16.61%, 14.64%; 62.81%, 28.18%, 4.60%; 37.50%, 27.08%, 31.90 respectively.

The intestine of *A*. *japonicus* had the highest bacterial richness on day 14 (Table 2), was indicated through the Ace and Chao1 peaks occurring on day 14. The Shannon index peaked on day 21, indicating that following immersion bathing in *L*. *plantarum* CLY-05, the intestine’s biodiversity had reached its highest level.

**Table 2:** Alpha diversity index of the intestine samples collected at different time.

Sample Code	Chao1 index	Ace Index	Shannon Index	Simpson Index
L0	326.67±9.68 ^a^	322.43±20.94 ^a^	4.37±0.47 ^ab^	0.90±0.04 ^ab^
L7	298.97±17.62 ^a^	302.66±9.49 ^ab^	3.65±0.06 ^a^	0.86±0.00 ^a^
L14	353.92±61.80 ^a^	328.06±45.85 ^a^	4.79±0.72 ^a^	0.92±0.04 ^a^
L21	318.30±33.66 ^a^	316.28±25.30 ^a^	4.86±0.28 ^a^	0.93±0.02 ^a^
L28	230.02±10.21 ^a^	226.34±18.14 ^a^	4.52±0.30 ^a^	0.91±0.02 ^a^
L35	266.06±41.60 ^ab^	262.43±45.14 ^ab^	4.01±0.57 ^ab^	0.81±0.07 ^a^
L42	268.94±50.12 ^ab^	262.70±44.45 ^ab^	4.22±0.63 ^ab^	0.87±0.04 ^ab^
L49	279.48±33.64 ^ab^	278.79±32.57 ^ab^	4.47±0.34 ^a^	0.91±0.04 ^ab^

Note: The different superscript letters within the same column mean significant differences (*P* < 0.05).

### 3.4 Correlation analysis between enzyme activities and the abundance of OTUs

**Fig 3:**
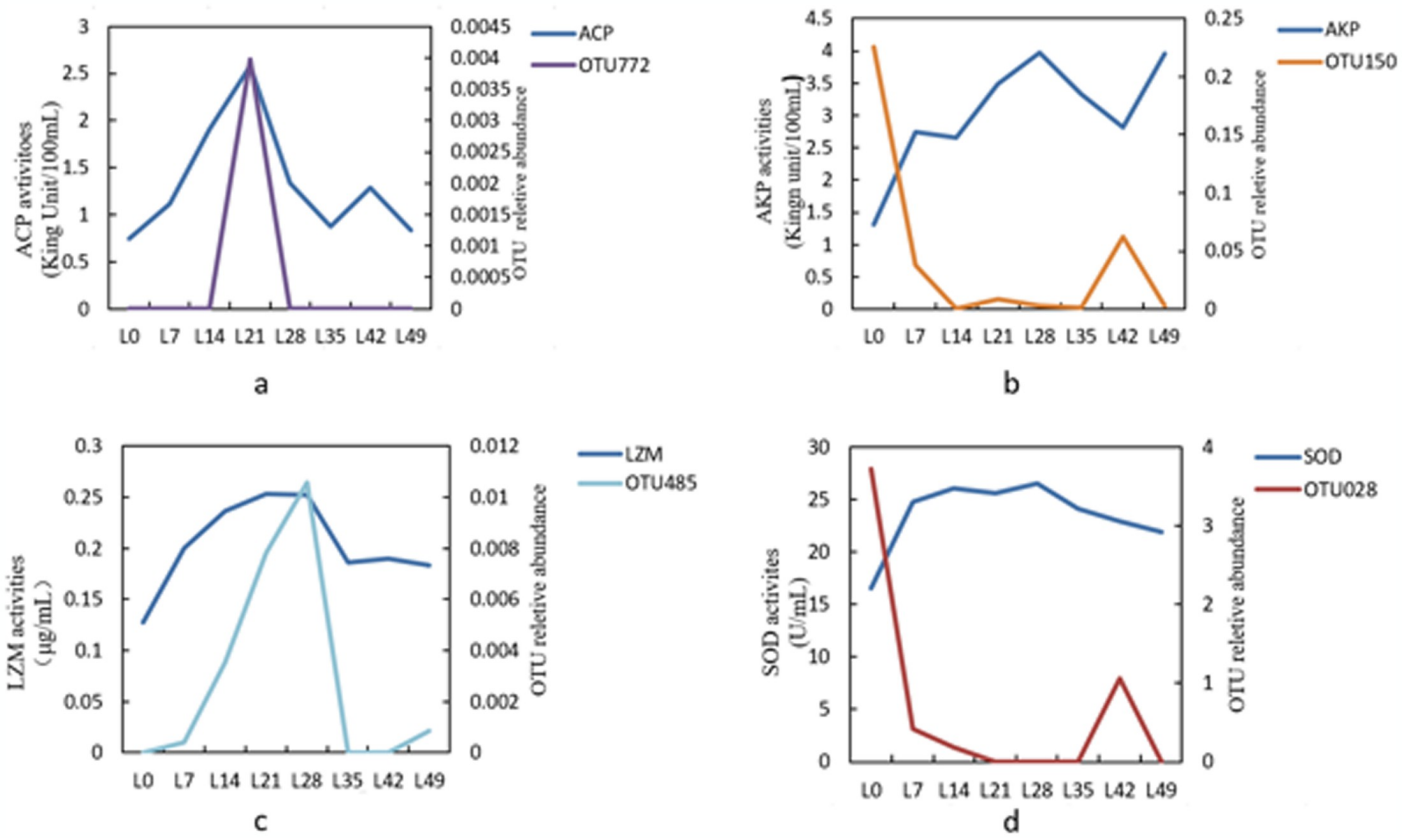
The correlation analysis between the selected OTUs and the change trend of non-specific immune enzyme activity.

In order to study the correlation between enzyme activities and the abundance of OTUs, specific OTUs were screened and a line chart of enzyme activities and OTUs was constructed (Fig 3), then, the specific OTUs are annotated (Table 3). A total of four OUTs were chosen (OTU772, OTU150, OTU485 and OTU028) that had a strong association with enzyme activity. With a Pearson correlation coefficient of 0.80, OTU772 showed a positive connection with ACP activity (*P < 0*.*05*). The Pearson correlation coefficient for OTU150 and AKP activity was -0.86, and the connection was negatively associated (*P < 0*.*01*). OTU485 and LZM activity were positively associated (*P < 0*.*05*), and the Pearson correlation coefficient was 0.81. The correlation between OTU28 and SOD activity is negative (*P <0*.*01*), with Pearson correlation coefficient value was -0.88. OTU772 was annotated to Proteobacteria, Deltaproteobacteria, Bdellovibrionales, Bacteriovoracaceae. OTU150 was annotated to Firmicutes, Bacilli, Bacillales, Bacillaceae. OTU485 was annotated to Proteobacteria, Gammaproteobacteria, Alteromonadales, Idiomarinaceae. OTU028 was annotated to Proteobacteria, Epsilonproteobacteria, Campylobacterales, Campylobacteraceae.

**Table 3:** Non-specific immune enzyme activities and its associated OTU screening and annotation results.

Name of enzyme activities	OUT-id	Pearson correlation	P-value	taxonomy			
				phylum	class	order	family
ACP	OTU772	0.80	0.016	Proteobacteria	Deltaproteobacteria	Bdellovibrionales	Bacteriovoracaceae
AKP	OTU150	-0.86	0.006	Firmicutes	Bacilli	Bacillales	Bacillaceae
LZM	OUT485	0.81	0.014	Proteobacteria	Gammaproteobacteria	Alteromonadales	Idiomarinaceae
SOD	OTU028	-0.88	0.004	Proteobacteria	Epsilonproteobacteria	Campylobacterales	Campylobacteraceae

## 4. Discussion

Lactic acid bacteria are the most commonly used probiotics, which can improve the nutritional value of feed, enhance host resistance to disease, or improve the quality of the ambient environment to modify the host associated or ambient microbial community [18, 19]. *L*. *plantarum* is a type of classical anaerobic lactobacillus bacteria in intestine [20], which has been reported of good probiotic properties [21, 22]. In this study, different concentrations of *L*. *plantarum* CLY-05 were added to the sea cucumber aquaculture water, and it turned out that the WGR of the experimental groups was higher than the control group, and the probiotic effect was related to the dosage. Those results were consistent with many previous studies [23–25]. *L*. *plantarum* is known to produce proteolytic and glycoside hydrolase enzymes that aid protein and carbohydrate digestion, therefore, improve the digestibility and nutritional value of feed [26–28]. This may be one of the reasons why *L*. *plantarum* promotes the growth of sea cucumbers.

It was reported that *L*. *plantarum* could accelerate the production of IgA and enhance the immunity level of the hosts [22, 29]. Chiu et al. [30] reported that administration of *L*. *plantarum* induced immune modulation, enhanced the immune ability, and increased resistance to *Vibrio alginolyticus* of *Litopenaeus vannam*ei. Valipour et al. [22] reported that the phenoloxidase (PO), lysozyme (LYZ), superoxide dismutase (SOD) and catalase (CAT) activity of narrow clawed crayfish (*Astacus leptodactylus*) were increased after dietary feeding of *L*. *plantarum* in 10^7^ or 10^8^ CFU/g. In this study, we also found that *L*. *plantarum* CLY-05 could improve the non-specific immunity of the sea cucumbers, so it is worthy studying the application of *L*. *plantarum* CLY-05 in the control of sea cucumber diseases.

The type, abundance and structure of intestinal flora play a decisive role in intestinal function. There were mounts of bacteria in the digestive tract, which develop an interdependence and mutual control relationship with host. Lactic acid bacteria could inhibit the growth and reproduction of the pathogen, and interact with gastrointestinal mucosa immunity system to maintain the microbiology balance in the gut [31]. Ramos et al. [32] found that fed *Oncorhynchus mykiss* with probiotics could improve the gut microbiota diversity. Purwandari et al. [33] found that the *Bacillus subtilis* could promote the body weight and improve the gut microbiota diversity of the *Epinephelus*. *coioides*. In this study, the usage of 1×10^3^ CFU/mL *L*. *plantarum* CLY-05 put the gut microbiota of sea cucumber into dynamic process. The richness and diversity of the gut microbiota peaked at the day 14 and day 21 after immersion bathing in *L*. *plantarum*. Therefore, *L*. *plantarum* affected the composition of the sea cucumber gut micrbiota in the relative abundance, which maybe the reason for growth and immunity improvement.

Studies in vitro cell, animal models and clinical trials had demonstrated that intestinal bacteria could maintain the integrity of intestinal barrier and affected the intestinal barrier function [34]. In this study, correlation analysis revealed that after immersion bathing in *L*. *Plantarum*, the change of non-specific immune enzyme activities of sea cucumbers had significantly correlation, and mainly focused on the phyla Firmicutes and Proteobacteria. Relevant studies have shown that some of the microorganisms in Firmicutes can promote the fermentation of carbohydrates and the decomposition of proteins, thus contributing to the healthy growth of organisms [35]. In this study, the association between gut microbiota and enzyme activity of *A*. *japonicus after* bathing in *L*. *plantarum* CLY-05 was analyzed, and 4 OTUs were screened to have a strong correlation with enzyme activity. The main pathogenic bacteria of ‘skin ulcer syndrome’ for *A*. *japonicus* which were *P*. *nigrifaciens*, *V*. *splendidus*, *V*. *parahaemolyticus*, and *V*. *alginolyticus*, were belong to the phyla of Proteobacteria. Lactic acid bacteria can inhibit the growth of pathogen by fostering healthy intestine [36, 37], this maybe be reason that the gut microbiota changed since the *L*. *plantarum* CLY-05 was added.

## 5. Conclusion

The results suggested that the ideal addition concentration of *L*. *plantarum* CLY-05 was 1×10^3^ CFU/mL, and that its addition could improve the specific growth rate, increase the non-specific immune enzyme activity, optimize the structure of the intestinal flora, and increase the diversity of the intestinal flora of *A*. *japonicus*. The results of this study provide scientific data support for the industrial application of *L*. *plantarum* in sea cucumber culture, and contribute to the industrial application of probiotics, so as to promote the development of sea cucumber culture industry.

## Supporting information

S1 Raw data (PZFX)

S2 Raw data (PZFX)

S3 Raw data (PZFX)

S4 Raw data (PZFX)

S5 Raw data (XLS)

S6 Raw data (XLSX)

S7 Raw data (XLSX)
